# *Crotalus atrox* venom preconditioning increases plasma fibrinogen and reduces perioperative hemorrhage in a rat model of surgical brain injury

**DOI:** 10.1038/srep40821

**Published:** 2017-01-19

**Authors:** Cherine H. Kim, Devin W. McBride, Ronak Raval, Prativa Sherchan, Karen L. Hay, Eric C. K. Gren, Wayne Kelln, Tim Lekic, William K. Hayes, Brian S. Bull, Richard Applegate, Jiping Tang, John H. Zhang

**Affiliations:** 1 Department of Physiology & Pharmacology, Loma Linda University School of Medicine, 11175 Campus St, Loma Linda, CA 92350, USA; 2 Department of Anesthesiology, Loma Linda University School of Medicine, 11175 Campus St, Loma Linda, CA 92350, USA; 3 Department of Earth and Biological Sciences, Loma Linda University School of Medicine, 11175 Campus St, Loma Linda, CA 92350, USA; 4 Department of Neurology, Loma Linda University School of Medicine, 11175 Campus St, Loma Linda, CA 92350, USA; 5 Department of Pathology and Human Anatomy, Loma Linda University School of Medicine, Loma Linda, California, USA; 6 Department of Neurosurgery Loma Linda University School of Medicine, 11175 Campus St, Loma Linda, CA 92350, USA.

## Abstract

Perioperative bleeding is a potentially devastating complication in neurosurgical patients, and plasma fibrinogen concentration has been identified as a potential modifiable risk factor for perioperative bleeding. The aim of this study was to evaluate preconditioning with *Crotalus atrox* venom (Cv-PC) as potential preventive therapy for reducing perioperative hemorrhage in the rodent model of surgical brain injury (SBI). *C. atrox* venom contains snake venom metalloproteinases that cleave fibrinogen into fibrin split products without inducing clotting. Separately, fibrinogen split products induce fibrinogen production, thereby elevating plasma fibrinogen levels. Thus, the hypothesis was that preconditioning with *C. atrox* venom will produce fibrinogen spilt products, thereby upregulating fibrinogen levels, ultimately improving perioperative hemostasis during SBI. We observed that Cv-PC SBI animals had significantly reduced intraoperative hemorrhage and postoperative hematoma volumes compared to those of vehicle preconditioned SBI animals. Cv-PC animals were also found to have higher levels of plasma fibrinogen at the time of surgery, with unchanged prothrombin time. Cv-PC studies with fractions of *C. atrox* venom suggest that snake venom metalloproteinases are largely responsible for the improved hemostasis by Cv-PC. Our findings indicate that Cv-PC increases plasma fibrinogen levels and may provide a promising therapy for reducing perioperative hemorrhage in elective surgeries.

Perioperative bleeding is a serious complication in patients undergoing neurosurgical procedures^1^,^2^. Intraoperative hemorrhage lengthens and complicates surgery and may trigger further injury by tissue hypo-perfusion or disruption of the blood-brain barrier^1^,^3^. Postoperative hemorrhage poses a significant threat, given the finite nature of the intracranial space. Even small hematomas may result in increased intracranial pressure and brain herniation. Thus, the optimization of perioperative hemostasis in neurosurgical patients is of paramount importance.

The surgical brain injury (SBI) rodent model has been established to mimic injury during brain resection^4^,^5^. Given the elective nature of most neurosurgical procedures, the SBI model is an ideal platform for preconditioning modalities, which are preemptive therapies in which mildly harmful stimuli are administered to induce endogenous protective mechanisms prior to major injury. Preconditioning has shown to be protective in the SBI model^6^,^7^, as well as in many other injury models^8^,^9^,^10^,^11^,^12^,^13^.

Fibrinogen, an acute phase reactant with a half-life of 3.74 days^14^, is part of the coagulation cascade and provides the substrate for fibrin clot formation. During coagulation, thrombin cleaves fibrinogen, producing soluble fibrin monomers that are able to be cross-linked by factor VIIIa to form a network on which a clot is built; fibrinogen also plays a role in platelet aggregation^15^. Decreased plasma fibrinogen levels have been identified as a potential modifiable risk factor for perioperative bleeding in cardiothoracic and orthopedic surgeries^16^,^17^,^18^,^19^. Lower perioperative fibrinogen levels, even within the normal reference range, have been shown to be associated with an increased risk of postoperative bleeding complications in patients undergoing elective intracranial surgery^20^,^21^. In current practice, the options for increasing plasma fibrinogen are limited to transfusions of fibrinogen-containing preparations, such as fresh frozen plasma, cryoprecipitate, and fibrinogen concentrate. The concentrations of fibrinogen in these preparations vary and large volumes of fresh frozen plasma are often necessary to increase plasma fibrinogen, making fluid overload an important consideration in fibrinogen supplementation. Furthermore, these transfusions are not without risk; they are associated with complications that include allergic reaction, infections, and transfusion-related acute lung injury^22^,^23^.

The hemostatic properties of snake venom toxins have long been studied for potential uses in clinical medicine. Some are used to detect coagulation disorders in the medical laboratory, while others have been examined for treating patients with coagulopathies^24^,^25^. *Crotalus atrox* venom contains snake venom metalloproteinases that cleave fibrinogen into fibrin split products in such a way that renders the proteins useless in fibrin polymerization^26^,^27^. Fibrin split products have been previously shown to increase fibrinogen plasma levels by inducing endogenous production of fibrinogen in hepatocytes^28^,^29^,^30^. The unique degradation of fibrinogen by *C. atrox* snake venom metalloproteinases —creating fibrin split products without the induction of clotting—presents an opportunity to harness the toxic nature of the venom to elicit an endogenous response to increase plasma fibrinogen levels prior to elective surgery. We hypothesize that *C. atrox* venom preconditioning (Cv-PC) prior to surgery will increase plasma fibrinogen, thereby decreasing perioperative bleeding in the rodent surgical brain injury model.

## Results

### Cv-PC Reduces Intraoperative Blood Loss *In Vivo*

**Figure 1:**
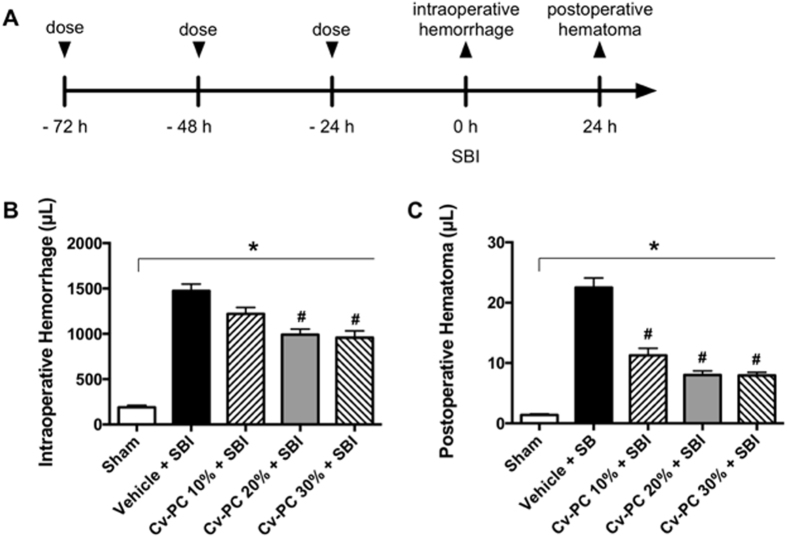
*Crotalus atrox* venom preconditioning (Cv-PC) reduces intraoperative hemorrhage and postoperative hematoma in a dose-dependent manner in SBI. (**A**) Schematic timeline of treatments and outcomes; treatment group rats received three subcutaneous doses of vehicle (normal saline) or Cv-PC (10%, 20%, and 30% of the LD_50_ dose) at 72 h, 48 h, 24 h prior to surgery; sham animals received craniotomy only; intraoperative hemorrhage volume was collected throughout surgery and postoperative hematoma was collected at 24 h post-surgery. (**B**) Intraoperative hemorrhage and (**C**) postoperative hematoma volumes were assessed by spectrophotometric hemoglobin assay; both were reduced by subcutaneous Cv-PC in a dose-dependent manner. Cv-PC: *Crotalus atrox* venom preconditioning; SBI: surgical brain injury. *p < 0.05 vs Sham, ^#^p <0 .05 vs Vehicle. Data are shown as mean ± SEM, n = 5–6 all groups. 1-way ANOVA, with Tukey’s comparisons, was used to determine differences.

SBI resulted in an intraoperative hemorrhage volume of 1475 ± 75 μL which was significantly higher than that observed in sham animals (188 ± 23 μL). Twenty-four hours after Cv-PC, a dose-dependent reduction of intraoperative hemorrhage by 17.2% to 35.0% was observed in Cv-PC animals subjected to SBI: 1221 ± 70 μL, 991 ± 62 μL, and 958 ± 75 μL in the 10%, 20%, and 30% LD_50_ (50% of the lethal dose) doses, respectively (Fig. 1B). Cv-PC with doses of 20% and 30% LD_50_ both significantly attenuated intraoperative hemorrhage compared to that of SBI animals given saline only (vehicle); however, the reduction in hemorrhage plateaued at the 20% LD_50_ level.

### Cv-PC Reduces Postoperative Hematoma *In Vivo*

Next, the effect of Cv-PC on the development of postoperative hematoma in the brain parenchyma was examined. Assessed at 24 h after SBI surgery, the postoperative hematoma demonstrated a dose-dependent decrease in volume by 48.7% to 64.9% for Cv-PC, which was significantly decreased for all doses compared to that of SBI animals receiving vehicle preconditioning (Fig. 1C). The postoperative hematoma volume in vehicle preconditioned animals was 22.5 ± 1.6 μL, and that of Cv-PC animals was 11.3 ± 1.2 μL, 8.0 ± 0.7 μL, and 7.9 ± 0.5 μL in the 10%, 20%, and 30% LD_50_ doses, respectively.

### Cv-PC Increases Plasma Fibrinogen and Plasma Fibrin Split Products in Rats

**Figure 2:**
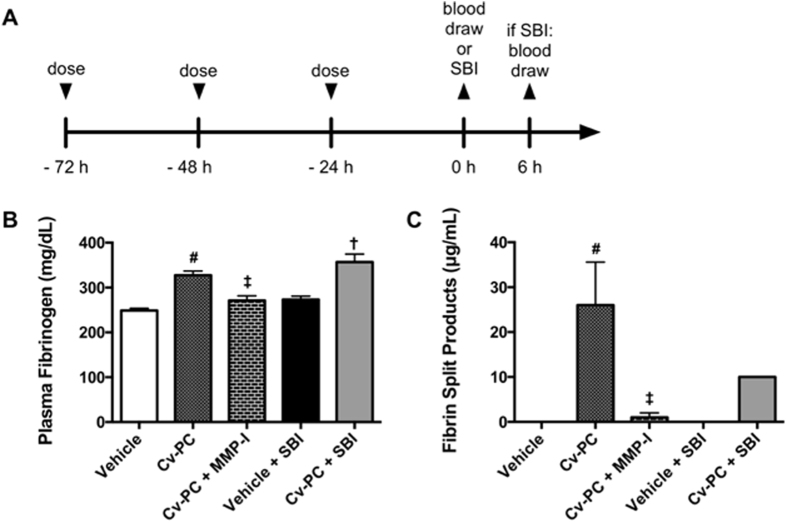
Cv-PC increases plasma fibrinogen and generates fibrin split products. (**A**) Schematic timeline of treatments and outcomes; rats received three subcutaneous injections of vehicle (normal saline), Cv-PC doses (20% of the LD_50_ dose), or Cv-PC doses incubated with matrix metalloproteinase inhibitor (MMP-I; with equal concentrations of Marimastat and AG-3340 to *C. atrox* venom) at 72 h, 48 h, 24 h prior to blood draw by cardiac puncture or surgery; animals undergoing surgery received blood draws at 6 h post-surgery. (**B**) Cv-PC significantly increased plasma fibrinogen at the time of surgery and at 6 h post-surgery; matrix metalloproteinase inhibition suppressed Cv-PC-induced increase in fibrinogen. (**C**) Fibrin split products were only detectable in the plasma of Cv-PC animals. Matrix metalloproteinase inhibition suppressed formation of fibrin split products by Cv-PC. ^#^p < 0.05 vs Vehicle, ^†^p < 0.05 vs Vehicle + SBI, ^‡^p < 0.05 vs Cv-PC. Data are shown as mean ± SEM, n = 8–12 all groups. 1-way ANOVA, with Tukey’s comparisons, was used to determine differences.

To assess the hematologic effects of Cv-PC, coagulation parameters were measured following administration of vehicle or Cv-PC in Naïve rats at 24 h following the third dose of *C. atrox* venom (corresponding to the intended surgery time) or at 6 h post-surgery in animals that received SBI (Fig. 2A). Naïve rats that received Cv-PC showed significantly increased plasma fibrinogen at time of surgery. Plasma fibrinogen was also elevated in rats at 6 h after SBI (Fig. 2B and Table 1). By the time of surgery, Cv-PC raised plasma fibrinogen by an average of 78.4 mg/dL—a 31.6% increase in concentration. At 6 h post-SBI, the fibrinogen remained higher in the Cv-PC animals than that of Vehicle preconditioned animals. Fibrin split products were only detected in the plasma of Cv-PC animals.

### Prothrombin Time, International Normalized Ratio, Partial Thromboplastin Time, D-Dimer, and Soluble Fibrin Remain Normal After Cv-PC in Rats

To assess Cv-PC’s potential for inducing coagulopathic events we measured prothrombin time, international normalized ratio, partial thromboplastin time, D-Dimer, and soluble fibrin. No clinically significant changes in these parameters were observed following Cv-PC in Naïve animals or rats after SBI with the exception of elevated soluble fibrin in the SBI + Cv-PC animals (Table 1).

### Preconditioning with Several C. atrox Fractions Reduces Intraoperative Hemorrhage and Postoperative Hematoma Volumes in the SBI Rat Model

**Figure 3:**
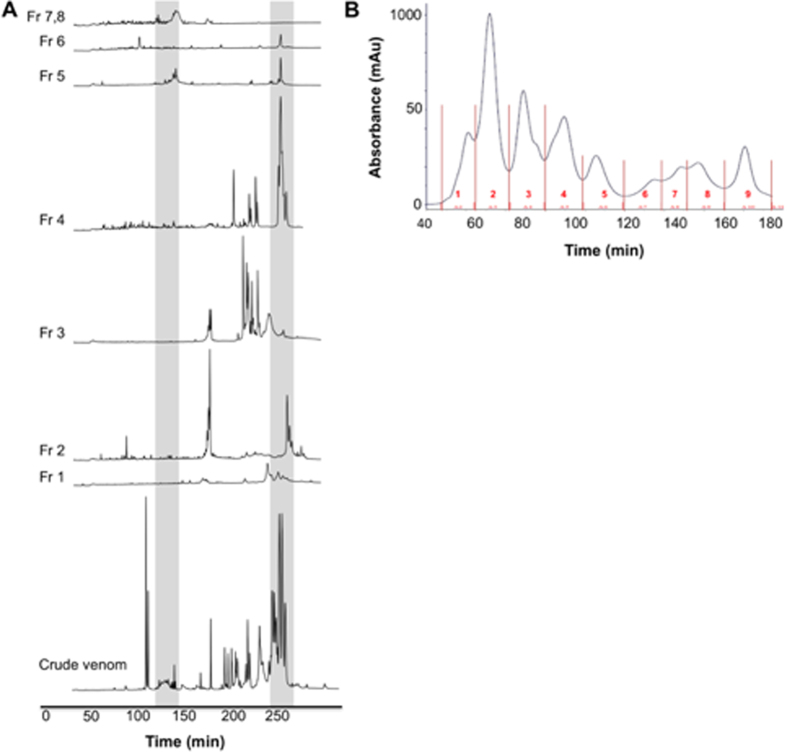
Reversed-phase HPLC and gel filtration chromatographs of *C. atrox* venom. (**A**) Reversed-Phase HPLC of crude *C. atrox* venom and *C. atrox* venom fractions (Fr) collected from gel filtration chromatography; absorbance at 214 nm. Shaded regions represent snake venom metalloproteinase components identified by MALDI mass spectrometry. (**B**) Gel Filtration Chromatograph of *C. atrox* venom fractionation. Collected fractions are labeled in red numbers.

Whole *C. atrox* venom and its fractions from gel filtration separation (Fig. 3B) were separated by reversed-phase HPLC (Fig. 3A), and fraction components of all substantial peaks were identified by LC-MS. A significant portion of the crude venom from *C. atrox* was identified as snake venom metalloproteinases (grey shading on Fig. 3A, also see Supplemental Fig. S1 and Supplemental Table S1). The peaks which correspond to snake venom metalloproteinases in each HPLC fraction is displayed with the grey shading (Fig. 3A). The gel filtration fractions 2–5 had the greatest concentrations of the snake venom metalloproteinases in the *C. atrox* venom.

**Figure 4:**
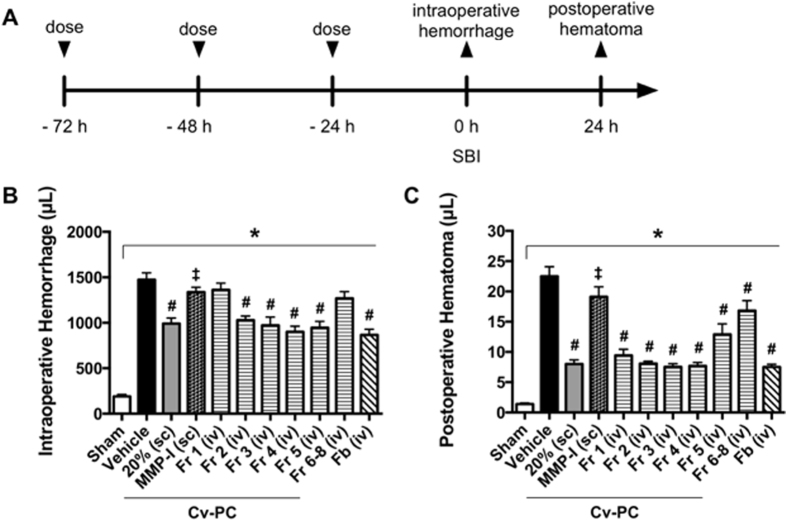
Cv-PC intraoperative hemorrhage and postoperative hematoma in SBI. (**A**) Schematic timeline of treatments and outcomes; rats received three subcutaneous doses of vehicle (normal saline), Cv-PC (20% of the LD_50_ dose), Cv-PC doses incubated with matrix metalloproteinase inhibitor (MMP-I; with equal concentrations of Marimastat and AG-3340 to *C. atrox* venom), or fractions (Fr) of *C. atrox* venom at 72 h, 48 h, 24 h prior to surgery. Sham animals received craniotomy only. Fibrinogen-treated animals received one dose of fibrinogen (Fb, 15 mg/1.5 mL) 15 min before surgery. Routes of dose administration were either subcutaneous (sc) or intravenous (iv). Intraoperative hemorrhage volume was collected throughout surgery and postoperative hematoma was collected at 24 h post-surgery. (**B**) Intraoperative hemorrhage volume was significantly reduced by Cv-PC (20% LD_50_) and Cv-PC by Fr 2, 3, and 4. Matrix metalloproteinase inhibition during Cv-PC reversed the effect. Direct administration of fibrinogen reduced intraoperative hemorrhage. (**C**) Postoperative hematoma volume was significantly decreased by Cv-PC (20% LD_50_) and Cv-PC by all fractions. Matrix metalloproteinase inhibition during Cv-PC reversed the effect. Direct administration of fibrinogen reduced the development of postoperative hematoma. *p < 0.05 vs Sham, ^#^p < 0.05 vs Vehicle, ^‡^p < 0.05 vs Cv-PC (20% LD_50_). Data are shown as mean ± SEM, n = 5–6 all groups. 1-way ANOVA, with Tukey’s comparisons, was used to determine differences.

To identify which protein(s) may be responsible for Cv-PC with *C. atrox* venom, the individual gel filtration *C. atrox* venom fractions 1, 2, 3, 4, 5 and pooled fractions 6–8 were used for separate Cv-PC regimens. Cv-PC with fractions 2, 3, 4, and 5 improved intraoperative hemorrhage volumes (Fig. 4A), while all fractions had marked reductions of the postoperative hematoma (Fig. 4B).

### Matrix Metalloproteinase Inhibition Blocks Cv-PC Effects on Hemorrhage Volumes, Plasma Fibrinogen and Fibrin Split Products in the Rat SBI Model

To evaluate the role of snake venom metalloproteinases in Cv-PC against SBI, two matrix metalloproteinase inhibitors were coadministered with *C. atrox* venom for each injection during Cv-PC. The concomitant administration of matrix metalloproteinase inhibitors abolished the increase in plasma fibrinogen by Cv-PC (Fig. 2B and Table 1) to levels similar to that of Vehicle preconditioned animals for both the naïve and SBI groups. Additionally, Cv-PC administered with matrix metalloproteinase inhibitors significantly diminished the generation of fibrin split products (Fig. 2C and Table 1). Furthermore, snake venom metalloproteinase inhibition by matrix metalloproteinase inhibitors had similar intraoperative hemorrhage and postoperative hematoma volumes to those of vehicle preconditioning animals; abolishing the protective effects of Cv-PC (Fig. 4B and C).

### Direct Fibrinogen Administration Reduces Intraoperative Hemorrhage and Postoperative Hematoma Volumes in the Rat SBI Model

To determine the effect of direct fibrinogen administration on SBI pathophysiology, 15 mg/1.5 mL of fibrinogen was administered 15 min prior to induction of surgery. In fibrinogen-treated animals, a reduction of intraoperative hemorrhage (Fig. 4B) and postoperative hematoma volumes (Fig. 4C) were observed comparable to those of Cv-PC animals.

### *C. atrox* Venom Causes Hemostatic Changes in Human Whole Blood

**Figure 5:**
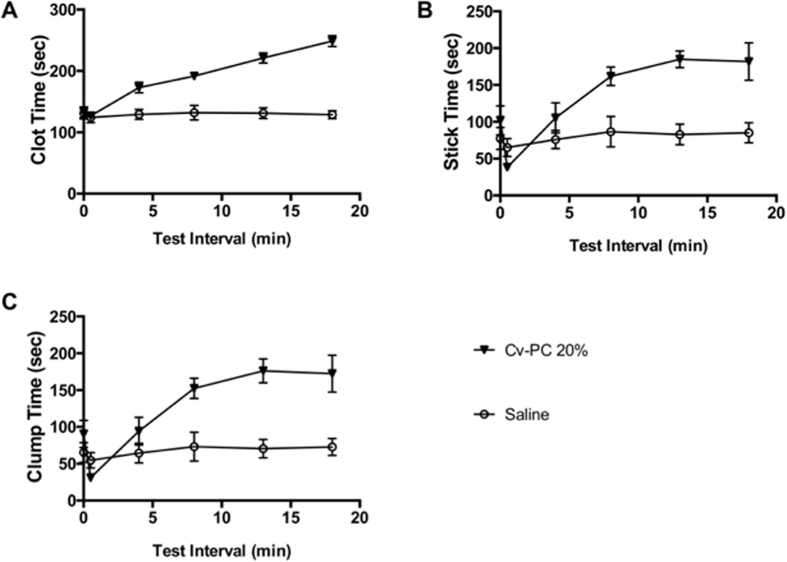
Effects of *C. atrox* venom on human whole blood *in vitro*. Minimal stimulation clotting/platelet testing imitates *in vivo* clotting on a sample of whole blood and measures three parameters—clot, clump, and stick times. Clot time correlates with fibrinogen, intrinsic/extrinsic pathway function, whereas clump and stick times correlate well with platelet function. *C. atrox* venom causes an increase in (**A**) clot time, (**B**) stick time, and (**C**) clump time. Data are shown as mean ± SEM, n = 3 all groups.

To begin assessing the clinical feasibility of Cv-PC, the direct effects of *C. atrox* venom on human whole blood was evaluated for clotting, sticking, and clumping parameters (Fig. 5). No change in any of these measures was found for saline; however, *C. atrox* venom has a dramatic effect on human whole blood. Initially, *C. atrox* venom caused a rapid decrease (for stick and clump times), which was then followed by an increase in all three parameters.

## Discussion

Perioperative hemorrhage is a devastating complication in neurosurgery^1^,^2^, making optimization of hemostasis of the utmost importance. This study uses a novel preemptive approach for improving perioperative hemostasis by eliciting endogenous mechanisms using *C. atrox* venom preconditioning. Our hypothesis was that Cv-PC would attenuate the hemorrhage induced by SBI via an increase of endogenous fibrinogen production. First, we observed that Cv-PC reduces intraoperative blood loss and postoperative hematoma by more than 30% in the SBI rodent model. Second, while coagulation was improved by Cv-PC, coagulation parameters—prothrombin time, international normalized ratio, partial thromboplastin time, D-Dimer, and soluble fibrin—showed no indication that Cv-PC caused a thrombotic state. Third, our results point to snake venom metalloproteinases as the active component of *C. atrox* venom that cleaves fibrinogen into fibrin split products and imparts the protective effects of Cv-PC; and inhibition of snake venom metalloproteinases reversed the effects of Cv-PC. Finally, preconditioning with *C. atrox* venom fractions, which primarily contain snake venom metalloproteinases, retained the effects of Cv-PC by crude venom.

Given the ability of *Crotalus* venoms to generate fibrin split products without the initiation of clotting^26^,^27^, and the previously demonstrated induction by fibrin split products of hepatocyte fibrinogen production^28^,^29^,^30^, *C. adamanteus, C. atrox,* and *C. viridis helleri* venoms were considered for preconditioning. We elected to use *C. atrox* venom because it has been demonstrated to have strong fibrinolytic activity with no detectable fibrinogen clotting activity, whereas the other *Crotalus* venoms were measured to have some fibrinogen clotting activity^31^. To determine the optimal dose of venom that can elicit therapeutic benefit to animals subjected to SBI, a dose dependent study was conducted in which we administered three daily doses of *C. atrox* venom (10%, 20%, and 30% of the LD_50_; 1.85 mg/kg, 3.7 mg/kg, and 5.55 mg/kg, respectively) with the last dose 24 h prior to surgery. The results of the dose dependent study suggest that the effectiveness of Cv-PC for reducing intraoperative hemorrhage and postoperative hematoma volumes plateaus at the 20% LD_50_ dose. Additionally, these data suggest that Cv-PC is a viable treatment to improve hemostasis in the SBI neurosurgical model.

Prothrombin time, international normalized ratio, and partial thromboplastin time are routinely assessed to evaluate the extrinsic and intrinsic pathways of the coagulation cascade. Increased international normalized ratio or prolongation of prothrombin time or partial thromboplastin time is suggestive of bleeding diathesis. Shortened partial thromboplastin time may be associated with increased risk of venous thromboembolism^32^. D-Dimer and soluble fibrin levels are used to screen for disseminated intravascular coagulation, which is characterized by systemic activation of blood coagulation and subsequent formation of microvascular thrombi that may ultimately lead to severe bleeding by consumptive depletion of coagulation factors and/or platelets^33^. The findings that Cv-PC had no clinically significant effect on any of these parameters supports previous findings that *C. atrox* venom has limited clotting activity^31^.

Initially, *C. atrox* venom was fractionated by reverse-phase chromatography, which may denature proteins, and is therefore unsuitable for assays of biological activity; thus, gel filtration chromatography, which preserves protein activity but achieves much lower resolution in protein separation than reversed-phase chromatography was utilized to obtain 9 fractions. These fractions were administered intravenously to assure intravascular exposure to the *C. atrox* venom components. In both intraoperative and postoperative hemorrhage volumes, fractions 2, 3, and 4, which contain primarily snake venom metalloproteinases (Fig. 3), provided improved hemostasis most consistently. The whole venom profile was dominated by a large peak complex eluting between 230 and 250 min, which aligned with major peaks in Gel-Filtration fractions 2–4 (Fig. 3A) MALDI protein identification indicated these peaks were composed entirely of snake venom metalloproteinases. Metalloproteinase fragments were also identified in several smaller peaks eluting between 215 and 245 min, which corresponded to peaks in Gel-Filtration fraction 5 and may explain the lesser Cv-PC activity also exhibited by this fraction.

To further elucidate snake venom metalloproteinases as the primary protein responsible for increased homeostasis during SBI, we evaluated the effects of blocking snake venom metalloproteinases during Cv-PC. Since specific inhibitors of snake venom metalloproteinases are not available, we elected to use commercially available matrix metalloproteinase inhibitors, Marimastat and AG-3340, which have been previously described to block other snake venom metalloproteinases^34^. Our findings, that coadministration of matrix metalloproteinase inhibitors and *C. atrox* venom during preconditioning prevents Cv-PC benefits, suggest that snake venom metalloproteinases plays a crucial role in the generation of fibrin split products and fibrinogen, as well as in the improved hemostasis conferred by Cv-PC.

To determine if fibrinogen was the key mediator of Cv-PC effect after SBI, fibrinogen treatment was given 15 minutes before SBI (15 mg/1.5 mL of fibrinogen). This dose was determined by computing the required amount of protein to raise plasma fibrinogen by approximately 75 mg/dL, given the estimated blood volume of the animals, as previously published^35^. Our results showed that this dose of fibrinogen, which correlates with the increase in fibrinogen concentration provided by Cv-PC, was capable of improving perioperative hemostasis.

Minimal stimulation clotting/platelet testing, which imitates *in vivo* clotting on a sample of whole blood, measures three parameters—clot, clump, and stick times. Clot time correlates with fibrinogen, intrinsic/extrinsic pathway function, whereas clump and stick times correlate well with platelet function. The increase in all three parameters by *C. atrox* venom demonstrates impaired clotting function. The prolonged clot time likely represents a depletion of fibrinogen by snake venom metalloproteinase cleavage. The prolonged clump and stick times indicate decreased platelet function, which likely results from fibrinogen depletion, as platelets are supported by fibrinogen to mediate adhesion and aggregation^36^,^37^,^38^.

Our findings are congruent with the growing body of literature that emphasizes plasma fibrinogen level as a potential modifiable risk factor for perioperative hemorrhage^16^,^17^,^18^,^19^,^20^,^21^. These studies indicate that lower fibrinogen, even within normal limits, are correlated with increased bleeding and that higher levels of fibrinogen are associated with better outcomes. Fibrinogen is fundamental to effective clot formation. It circulates at the highest concentration of all the coagulation factors and is the first coagulation factor to drop to critically low levels during major hemorrhage^39^. Dilutional deficiency of fibrinogen develops earlier than any other hemostatic abnormality when plasma-poor red blood cell preparations are used to replace blood loss^40^. Yet in current practice, the options for increasing plasma fibrinogen are limited to transfusions of fibrinogen-containing preparations like fresh frozen plasma, cryoprecipitate, and fibrinogen concentrate. Fresh frozen plasma varies in fibrinogen concentration as it is collected from donors. The concentration of fibrinogen in fresh frozen plasma reportedly ranges between 100–300 mg/dL^41^. Because of these concentrations, large volumes of fresh frozen plasma are necessary for even modest increases in plasma fibrinogen. Cryoprecipitate, obtained by concentrating fresh frozen plasma, has about 200 mg of fibrinogen per unit at a concentration of about 550 mg/dL^42^. Fibrinogen concentrate, manufactured from human plasma and available as a pasteurized, lyophilized powder, is given at a concentration of 2000 mg/dL^43^. A previous study determined that in order to raise plasma fibrinogen levels from 150 mg/dL to 170 mg/dL, requires 14 units of fresh frozen plasma, 8 units of cryoprecipitate, or 2 units of fibrinogen concentrate making fluid overload an important consideration in fibrinogen supplementation. In addition, these transfusions are not without risk; they are associated with complications that include allergic reaction, infections, and transfusion-related acute lung injury^22^,^23^.

An estimated 31% of elective neurosurgeries require blood transfusions^44^. A preventive therapy that reduces perioperative hemorrhage would decrease the need for blood transfusions, ultimately cutting perioperative costs; up to $6.03 million is spent on blood and transfusion-related care for surgical patients annually per hospital^45^. While our study focused on Cv-PC in the SBI model, the implications of our results extend beyond the field of neurosurgery. Cv-PC could also be applied to elective surgeries in other fields, as perioperative hemorrhage is the bane of surgeons in every specialty. Over 200 million major elective surgeries are performed worldwide per year^46^.

### Limitations and Future Studies

There are several limitations of this study. First, the *C. atrox* venom preconditioning regimen was arbitrarily selected. Herein, we used three days of single injections to precondition against SBI. Future studies will need to evaluate additional preconditioning regimens to determine the optimal dosing required to promote a significant rise in plasma fibrinogen levels. Second, *C. atrox* venom contains numerous proteins which are known to have hemostatic properties, including anti- and pro-coagulation. Here, we used gel filtration followed by HPLC to fractionate the crude *C. atrox* venom. However, the vast number of proteins in the crude venom required us to utilize pooled venom fractions (from gel filtration) to make the study manageable. Future studies will investigate the HPLC fractions of the gel filtration fractions which were capable of preconditioning against SBI and to establish a mechanism of protection (i.e. further fractionation of gel filtration fractions 2–5). Third, while our study demonstrated that Cv-PC did not result in coagulation parameters indicative of a thrombotic state, more studies are needed to assess whether the Cv-PC increases the risk of pathologic thrombotic events. Finally, additional studies are needed to assess the translational feasibility of Cv-PC and its safety.

## Methods

### Animal Experiments

All animal experiments were approved by the Institutional Animal Care and Use Committee at Loma Linda University and conducted in compliance with the *NIH Guidelines for the Use of Animals in Neuroscience Research*. Animals were housed in cages on a constant 12-h light/dark cycle in a controlled temperature room and were given food and water ad libitum. One hundred twenty-one male Sprague Dawley rats (280–330 g) were used in this study.

### Groups

The effects of Cv-PC on coagulation parameters as a therapy for SBI was investigated using naïve and SBI rats. Naïve animals were preconditioned with either the vehicle (normal saline), *C. artrox* venom, or *C. atrox* venom with matrix metalloproteinase inhibitors. SBI animals were divided in four groups: sham, vehicle preconditioning, Cv-PC, and fibrinogen treatment.

### Interventions – Naïve Animals

Vehicle preconditioned and Cv-PC animals received a single subcutaneous dose of *C. atrox* venom (or normal saline for the vehicle group) each day for three consecutive days before sacrifice (with the final dose 24 hours before sacrifice). A 20% LD_50_ dose of *C. atrox* venom was used (3.7 mg/kg). In Cv-PC + matrix metalloproteinase inhibitor animals, *C. atrox* venom (20% LD_50_) was incubated with Marimastat (3.7 mg/kg, Sigma) and AG-3340 (3.7 mg/kg, Sigma) for 30 min at 37 °C prior to subcutaneous injection^34^,^47^. The mixture of the crude venom and matrix metalloproteinase inhibitors was administered each day for three consecutive days before sacrifice.

### Interventions – SBI Animals

Vehicle preconditioned and Cv-PC animals received a single subcutaneous dose of *C. atrox* venom (or normal saline) each day for three consecutive days before SBI (with the final dose 24 hours before SBI). In the dose study (Fig. 1), *C. atrox* venom was administered at 10%, 20%, and 30% of the LD_50_ (1.85 mg/kg, 3.7 mg/kg, and 5.55 mg/kg, respectively). For the remainder of the experiments, the 20% LD_50_ dose of *C. atrox* venom was used; for Cv-PC conducted with Cv fractions, the same 3.7 mg/kg dosing was applied. Fibrinogen-treated animals received one dose of fibrinogen (1.5 mL, 10 mg/mL, Sigma) 15 min before SBI.

To identify the venom protein(s) responsible for the hemostatic effects, crude *C. atrox* venom was fractionated (as described below). Each fraction of the *C. atrox* venom was reconstituted in normal saline. The dose of each fraction was equivalent to its mass concentration of the crude venom (at 20% LD_50_). Preconditioning for each *C. atrox* venom fraction was performed via single IV injections each day for three consecutive days before SBI.

### Surgical Brain Injury Model

Rats underwent craniotomy and a partial right frontal lobe resection 1 mm above the horizontal line from bregma and 2 mm to the right of the vertical line from bregma down to the skull base as previously described^4^,^5^. After bleeding stopped, animals were sutured and allowed to recover. Sham animals underwent all surgical procedures with the exception of brain resection.

### Spectrophotometric Assay of Hemoglobin Volume

At 24 h after SBI, the animals were placed under deep anesthesia and transcardially perfused with 0.1 M PBS until the outflowing fluid from the right atrium became colorless. The brain was removed and dissected into left and right hemispheres. Hematoma volume was quantified by spectrophotometric assay of brain hemoglobin content as described previously^48^. A standard curve was obtained by adding incremental volumes of homologous blood to perfused brain tissue of naïve animals. The hemispheric samples were then homogenized and sonicated in distilled water followed by a 30-min centrifugation (13,000 *g* at 4 °C); Drabkin reagent (1.6 mL; Sigma) was added to 0.4 mL supernatant aliquots and optical density was measured at 540 nm via a spectrophotometer (Spectronix 3000; Milton-Roy). Hemoglobin measurements were compared with the standard curve to obtain hemorrhage volume expressed as μL of blood per ipsilateral hemisphere.

Intraoperative hemorrhage volume was collected by suction throughout surgery and was added to packing material used for hemostasis during the procedure; distilled water was added bringing the volume to 50 mL for each collected hemorrhage sample. A standard curve was obtained by adding incremental volumes of homologous blood to distilled water to create solutions of 50 mL total volume. Samples were homogenized, sonicated, and prepared for spectrophotometric assay as described above. Testing was done in duplicate.

### Blood Draws

6 h after SBI, animals were anesthetized with isoflurane and blood was collected by cardiac puncture using an 18-gauge needle; the first few drops of blood were discarded to eliminate tissue factor from the blood draw. Collected blood rapidly transferred to the appropriate collection tubes, gently mixed by ten inversions, and delivered to the appropriate laboratories for subsequent testing.

Specimens destined for soluble fibrin, prothrombin time, international normalized ratio, partial thromboplastin time, fibrinogen and D-Dimer testing were anticoagulated in a mixing ratio of one part citrate to nine parts blood using Greiner Bio-One Vacuette^®^ tubes (Part No. 454334) containing 3.2% (0.109 M) Sodium Citrate Solution. Specimens for Fibrin Split Product testing were added to Thrombo-Wellcotest™ Collection Tubes (Part No. R30853001) containing soy bean trypsin inhibitor (approximately 3600 NF units/tube) and *C. atrox* venom (>10 μg/tube) for the collection of each 2 mL of whole blood.

Previous studies have established normal ranges in rats using human test methods^33^.

### Soluble Fibrin Analysis

All samples were maintained at 37 °C from draw through testing. The testing was as described by Hay *et. al.,* but modified slightly since the appearance of endpoints in rat blood were somewhat atypical as compared to human endpoints^49^. The modification (reading endpoints using 90 μL plasma rather than the usual 150 μL whole blood) clarified the endpoints and allowed for comparison between rats and treatment groups. However, the results cannot be compared to usual human values.

### Prothrombin Time, International Normalized Ratio, Partial Thromboplastin Time, and Fibrinogen Analysis

Blood was centrifuged at 7200 RPM (4440 × g) for 2 min using the STATSpin Express 3 to obtain plasma for testing. Testing was performed using a BCS^®^ XP System (Siemens Healthcare, Munich, Germany). The prothrombin time and international normalized ratio were performed using Dade^®^ Innovin^®^ reagent. The partial thromboplastin time was performed using Dade^®^ Actin^®^ FSL reagent and 0.025 M CaCl_2_. Fibrinogen assays were performed using Dade^®^ Thrombin Reagent, Dade^®^ Fibrinogen Standard and Dade^®^ Owren’s Veronal Buffer.

### D-Dimer Analysis

Blood was centrifuged at 7200 RPM (4440 × g) for 2 min using the STATSpin Express 3 to obtain plasma for testing. Testing was performed using the Cobas^®^ 8000 modular analyzer (Roche Diagnostics, Indianapolis, IN). The method is based on latex particles coated with monoclonal anti-human D-Dimer antibodies (mouse) to which addition of a sample containing D-Dimer increases turbidity leading to changes in absorbance over time.

### Fibrin Split Products Analysis

Blood was centrifuged at 7200 RPM (4440 × g) for 2 min using the STATSpin Express 3 to obtain serum for testing. Testing was performed using the Thrombo-Wellcotest™ Rapid Latex FDP Assay. The kit test utilizes latex particles, which have been coated with rabbit antibodies to fibrin fragments. The approximate concentration is determined from agglutination observed on serial specimen dilutions.

### HPLC of *C. atrox* Venom

Lyophilized crude venom was diluted to a concentration of 6 mg/mL in Buffer A (0.065% TFA, 2% acetonitrile in Nanopure water) and centrifuged at 15,000 g for 10 min. 100 μL of the supernatant was fractionated on an ÄKTAmicro high-pressure liquid chromatography system (GE Healthcare Life Sciences, Piscataway, NJ, USA) fitted with two reversed-phase (RP) columns (SOURCE 5RPC ST polystyrene/divinylbenzene, 4.6 150 mm; GE Healthcare) run in series at a flow rate of 0.5 mL/min, using a linear gradient of 0–100% Buffer B (0.05% TFA, 80% acetonitrile in Nanopure water) over 40 column volumes. Protein elution was monitored at 214 nm using Unicorn 5.0 (GE Healthcare Lifesciences) software, and fractions were collected manually.

### Protein Identification

Each Reversed-Phase fraction was subjected to reduction and alkylation using dithiothreitol and iodoacetamide, respectively. Proteins were then digested with proteomics-grade porcine pancreatic trypsin (Sigma-Aldrich, St. Louis, MO). Samples were desalted using C18 ZipTips (EMD Millipore, Billerica, MA) according to the manufacturer’s protocol and submitted the samples to Shimadzu Scientific Instruments, Inc. (Columbia, MD) for peptide identification by Matrix-Assisted Laser Desorption/Ionization (MALDI). Because Reversed-Phase HPLC may denature proteins, these fractions were not used in the physiological activity assays. Instead, venom for Cv-PC use was fractionated via Gel Filtration chromatography using a Superdex gel filtration column (HiLoad 16-/60 Superdex 75PG, 17–1068–02, GE Healthcare) on an Amersham Biosciences ÄKTA FPLC (18–1900–26, GE Healthcare). Crude venom (4 mg/mL in 0.15 M ammonium bicarbonate) was injected into the column (500 mL sample) and separated in 0.15 M ammonium bicarbonate at a flowrate of 1 mL/min. Individual fractions were collected manually at the local minimums of each peak (based on absorbance at 214 nm). All collected fractions were lyophilized and stored at −20 °C until use. The protein concentration of each reconstituted fraction was measured using a NanoDrop 2000 UV-Vis Spectrophotometer (Thermo Scientific). Although Gel Filtration chromatography preserves protein activity, it fails to achieve the level of protein separation required for mass spectrometry. Therefore, to determine which venom components may explain the observed Cv-PC activity, each Gel Filtration fraction was subjected to Reversed-Phase HPLC, and the resulting partial venom profiles aligned against the whole venom Reversed-Phase chromatogram whose peaks were identified by mass spectrometry (Fig. 3A).

### Minimal Stimulation *In Vitro* Clotting/Platelet Testing

Consenting normal adults donated 5 mL of blood for each agent to be tested. Blood was drawn into citrated syringes (1 part citrate + 9 parts blood) and mixed gently, then all air was expressed, and a parafilm seal was placed over the luer tip. Syringes were maintained horizontally at 37 °C for approximately 50–60 min, allowing endothelial inhibitors to dissipate and platelet function to reach a stable phase. During this stable phase, multiple tests could be performed and the results compared.

Baseline platelet testing was done in duplicate at 37 °C as follows. Pre-warmed reaction tubes containing 1 mg Celite 270 (Manville Products, Denver, CO), 450 μL of 0.0055 M isotonic CaCl2 and 25–35 solid glass beads, 0.5 mm (#030001, Propper Manuf Co, NY) were pre-mixed. To this 3 drops (~150 μL) of well-mixed blood were added and time to completion of the clumping, sticking and clotting visual endpoints was measured on the Hemostasis Mechanism Analyzer (Boehringer Laboratories, Norristown, PA).

When done with baseline testing, the blood level in each syringe was adjusted to exactly 3 mL in preparation for testing of the various venom concentrations. After remixing, 10 μL of the venom was carefully added midway in the column of 3 mL whole blood using a Drummond pipette. After 30 s, the sample was remixed and testing was performed as described above. Testing was repeated periodically over ~20 min to monitor changes in platelet and clotting function after exposure to the venom.

### Statistical Analysis

All values are mean ± SEM. GraphPad Prism software was used for statistical analysis. We compared treatment groups for each dependent measure using a one-way analysis of variance (ANOVA) followed by Tukey’s multiple comparisons. A *p* value < 0.05 was considered significant.

### Study Approval

All animal husbandry and procedures were approved by the Institutional Animal Care and Use Committee. The study protocol was approved by the Institutional Review Board at Loma Linda University.

## Additional Information

**How to cite this article:** Kim, C. H. *et al. Crotalus atrox* venom preconditioning increases plasma fibrinogen and reduces perioperative hemorrhage in a rat model of surgical brain injury. *Sci. Rep.* **7**, 40821; doi: 10.1038/srep40821 (2017).

**Publisher's note:** Springer Nature remains neutral with regard to jurisdictional claims in published maps and institutional affiliations.

## Supplementary Material

Supplemental Information

## Figures and Tables

**Table 1:** Coagulation parameters after *Crotalus atrox* venom preconditioning.

	Naïve			SBI	
	Vehicle	Cv-PC	Cv-PC + MMP-I	Vehicle	Cv-PC
n =	12	10	10	10	8
Fibrinogen (mg/mL)	248.9 ± 4.9	327.6 ± 9.1*	271.0 ± 10.6	273.2 ± 7.6	356.9 ± 17.6*
Fibrin Spilt Products (μg/mL)	0.0 ± 0.0	26.0 ± 9.6*	1.0 ± 1.0	0.0 ± 0.0	10.0 ± 0.0*
Prothrombin Time (s)	10.67 ± 0.05	10.52 ± 0.05	10.34 ± 0.08	10.25 ± 0.10	10.04 ± 0.07
International Normalized Ratio	0.97 ± 0.01	0.92 ± 0.01	0.93 ± 0.01	0.91 ± 0.01	0.90 ± 0.00
Partial Thromboplastin Time (s)	15.07 ± 0.15	14.76 ± 0.04	15.32 ± 0.38	15.16 ± 0.12	14.83 ± 0.04
D-Dimer (μg/mL)	0.03 ± 0.01	0.00 ± 0.00	0.04 ± 0.02	0.01 ± 0.01	0.06 ± 0.01
Soluble Fibrin (ng/mL)	41.8 ± 8.9	65.6 ± 8.1	48.2 ± 10.2	39.5 ± 6.7	80.8 ± 11.3*

Data are shown as mean ± SEM. Cv-PC, *C. atrox* venom preconditioning; MMP-I, matrix metalloproteinase inhibitor; SBI, surgical brain injury. *p < 0.05 vs Vehicle.
