# Supplementary material for: Crotalus atrox venom preconditioning increases plasma fibrinogen and reduces perioperative hemorrhage in a rat model of surgical brain injury
Source: Sci Rep. 2017 Jan 19;7:40821. doi: 10.1038/srep40821 (PMC5244360; doi:10.1038/srep40821)
Supplement: Supplemental Information [file srep40821-s1.pdf]

Online Supplemental

Crotalus atrox venom preconditioning increases plasma fibrinogen and reduces perioperative hemorrhage in a rat model of surgical brain injury

Cherine H. Kim,[1] Devin W. McBride,[1] Ronak Raval,[2] Prativa Sherchan,[1] Karen L. Hay,[2] Eric C.K. Gren,[4] Wayne Kelln,[4] Tim Lekic,[1,5] William K. Hayes,,[4] Brian S. Bull,[6] Richard Applegate II,[2] Jiping Tang,[1] and John H. Zhang,[1,2,3]

[1]Department of Physiology & Pharmacology, [2]Department of Anesthesiology, [3]Department of Neurosurgery, [4]Department of Earth and Biological Sciences, [5]Department of Neurology, [6]Department of Pathology and Human Anatomy, Loma Linda University School of Medicine, Loma Linda, California, USA

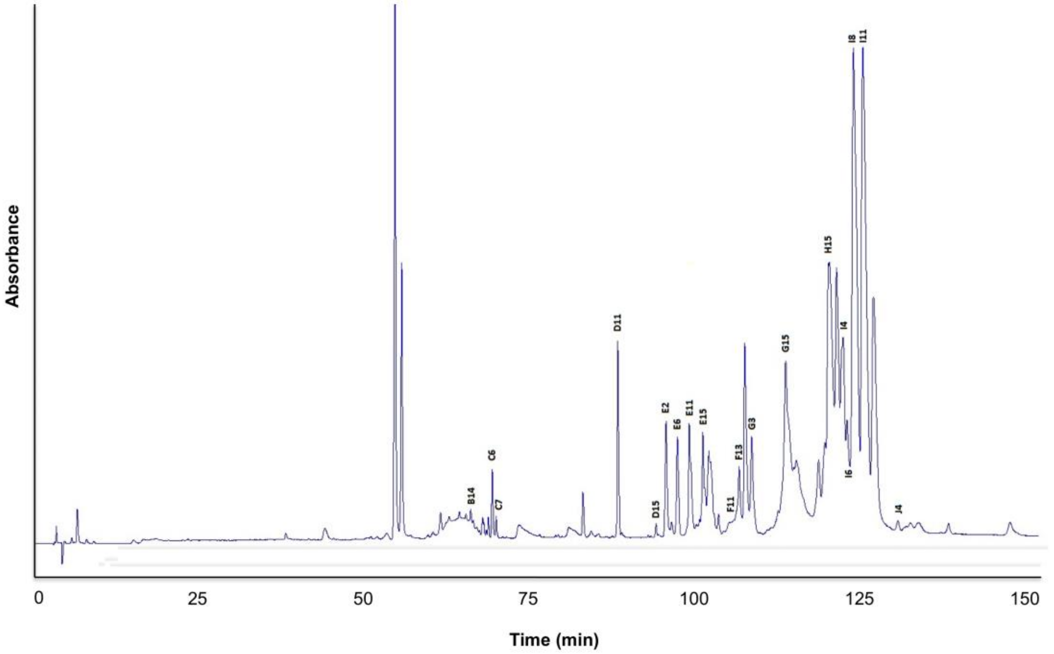

Supplementary Figure S1. Reversed-phase high pressure liquid chromatography separation of whole *C. atrox* venom. Labeled peaks are identified in Table S1.

Supplementary Table S1. Results of Matrix-Assisted Laser Desorption/Ionization (MALDI) protein identification

Fraction	Identification	Swiss Prot ID	Score	Unique peptides	Sequence
**B14**	Zinc metalloproteinase/disintegrin	VM2A_CROT	42	1	R.VSMVDRNDDTCTGQSADCPR.N
**C6**	Zinc metalloproteinase/disintegrin	VM2AE_CROAT	43	1	R.VSMVDRNDDTCTGQSADCPR.N
**C7**	Zinc metalloproteinase/disintegrin	VM2AE_CROAT	72	1	R.VSMVDRNDDTCTGQSADCPR.N + Deamidated (NQ)
**C7**	Zinc metalloproteinase/disintegrin	VM2AE_CROAT	56	2	K.MRPGSQCAEGLCCDQCR.F
					R.VSMVDRNDDTCTGQSADCPR.N
**D11**	Phospholipase A2 homolog Cax-K49	PA2H_CROAT	44	1	NPITSYGIYGCNCGVGSR
**D15**	Snake venom metalloproteinase atrolysin-B	VM1AB_CROAT	64	2	R.AYTSSMCNPR.K
					R.GASLCIMRPGLTPGR.S
**E2**	Catrin-1/2 OS=Crotalus atrox PE=1 SV=1	CRVP_CROAT	148	3	QMQSDCPAICFCQNK
					K.YSYFYVCQYCPAGNIIGK.T
					K.YGIGAVPPNAVTGHFSQVVWYK.S
**E6**	Phospholipase A2, basic isoform OS=Deinagkistrodon acutus	PA2B_DEIAC	95	2	K.NAFPFYTSYGCYCGWGGR.G
					R.KNAFPFYTSYGCYCGWGGR.G
**E11**	Snake venom serine protease catroxase-2 OS=Crotalus atrox PE=1 SV=1	VSP2_CROAT	70	1	LLD DAVCQPPYPELPATSR
**E15**	Snake venom serine proteinase Sp1	VSP1_CROAD	56	1	R.AAYPEYGLPATSR.T
**F11**	Galactose-specific lectin	LEGG_CROAT	103	3	K.GQENVWIGLR.D
					K.TWEDAEMFCR.K
					K.EFCVELVSLTGYR.L
**F13**	Galactose-specific lectin OS=Crotalus atrox PE=1 SV=1	LEGG_CROAT	66	1	R.YGESLEIAEYISDYHKGQENVWIGLR.D
**G3**	Phospholipase A2	PA2_CROT	45	1	R.SGLLWYSAYGCYCGWGGHGLPQDATDR.C
**G15**	L-amino-acid oxidase OS=Crotalus adamanteus PE=1 SV=1	OXLA_CROAD	61	1	IYFAGEYTAQFHGWIDSTIK
**H15**	Zinc metalloproteinase/disintegrin	VM2AE_CROAT	329	4	R.YVELFIVVDHGMYTK.Y
					K.SHDNAQLLTSIAFDEQIIGR.A

					R.KSHDNAQLLTSIAFDEQIIGR.A
					K.YFSDCSYIQCWEFIMNQKPQCILK.K
**I4**	Snake venom metalloproteinase atrolysin-C	VM1AC_CROAT	62	2	R.VHEIVNFINGFYR.S
					R.SYEFSADSMHYYER.F
**I6**	Snake venom metalloproteinase HT	VM1H2_CRORU	45	1	R. VHEIVNFINEFYR. S
**I8**	Snake venom metalloproteinase	VM1H2_CRORU	41	1	R.VHEIVNFINEFYR.S
**I11**	Zinc metalloproteinase-disintegrin VMP-III OS=Crotalus viridis viridis PE=2 SV=1	VM3V3_CROVV	192	2	R.MYEIVNTVNEIYR.Y
					K.ITVKPEAGYTLNAFGEWR.K
**J4**	Zinc metalloproteinase/disintegrin VMP-II	VM2V2_CROVV	47	1	R.IWVHEIVNTINVFYR.S
